# Treatment strategy for septic arthritis of the sternoclavicular joint with osteomyelitis, large abscesses, and mediastinitis: A case report

**DOI:** 10.1016/j.ijscr.2025.111986

**Published:** 2025-09-25

**Authors:** Ryo Maeda, Ryusei Yamada, Mayu Inomata, Fumiaki Kawano

**Affiliations:** a Department of Thoracic and Breast Surgery, Faculty of Medicine, University of Miyazaki, Miyazaki, Japan; b Department of Surgery, Faculty of Medicine, University of Miyazaki, Miyazaki, Japan

**Keywords:** Case report, Surgery, Septic arthritis of the sternoclavicular joint, Omental flap, Osteomyelitis

## Abstract

**Introduction and importance:**

Septic arthritis of the sternoclavicular joint (SASCJ) accounts for <1 % of all joint infections. Owing to the limited literature on the disease, there is no consensus on the general treatment for SASCJ. Here, we report a case of SASCJ complicated by osteomyelitis, mediastinitis, infectious myositis, and cervical and chest wall abscesses that required radical sternoclavicular joint resection and reconstruction using the greater omental flap. In this report, we discuss the treatment strategy for advanced SASCJ cases.

**Case presentation:**

A 65-year-old man was diagnosed with SASCJ relapse after the failure of antibiotic therapy alone, combined with osteomyelitis, mediastinitis, infectious myositis, and cervical and chest wall abscesses. A radical sternoclavicular joint resection was performed. After negative-pressure wound therapy, serial re-debridement was performed. The greater omental flap was transported into a deep, large residual space. The patient has been relapse-free for 1 year.

**Clinical discussion:**

Radical resection of the entire sternoclavicular joint should be the preferred management strategy for patients with sternoclavicular joint infections, especially in cases of osteomyelitis. Although the pectoralis major flap is typically the first choice to cover the defect, we used the omental flap because the locoregional flaps were not large enough to reconstruct the defect.

**Conclusion:**

The greater omental flap is an advantageous alternative when there is a need to provide coverage for large defects in cases of SASCJ.

## Highlights

• There is no consensus on the general treatment for septic arthritis of the sternoclavicular joint
• A treatment strategy for advanced cases of septic arthritis of the sternoclavicular joint
• Usefulness of the greater omental flap for reconstruction

## 1 Introduction

The sternoclavicular joint (SCJ) is a synovial saddle joint between the manubrium and clavicle. Septic arthritis of the SCJ (SASCJ) accounts for <1 % of all joint infections [1]. Delayed diagnosis and inappropriate treatment may lead to the spread of bacterial infection to adjacent structures, leading to potentially life-threatening complications, such as chest wall abscess [2] and mediastinitis [3]. Owing to the limited literature on the disease, there is no consensus on the general treatment for septic arthritis. Treatment strategies range from radical surgical debridement [3,5] to conservative treatment with antibiotics alone [6], depending on the clinical presentation.

Here, we report a case of SASCJ complicated by osteomyelitis, mediastinitis, infectious myositis, and cervical and chest wall abscesses that required radical SCJ resection and reconstruction using the greater omental flap. In this report, we discuss the treatment strategy for advanced SASCJ cases. This study was conducted following the principles of the Declaration of Helsinki and the SCARE guidelines [7].

## 2 Case presentation

**Fig. 1:**
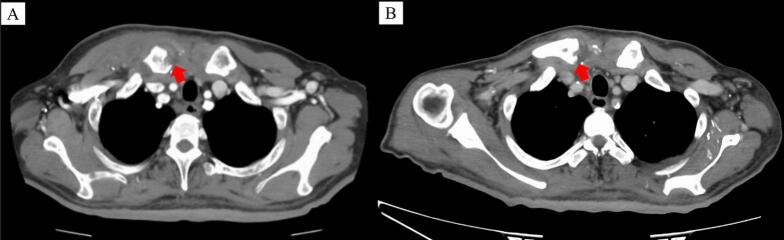
(A) Computed tomography shows erosions (a red arrow) of the right sternoclavicular joint. (B) Erosions (red arrows) remain at the last dose of vancomycin. (For interpretation of the references to colour in this figure legend, the reader is referred to the web version of this article.)

A 65-year-old man presented with a subcutaneous mass in the right anterior chest region. Three weeks prior, he had received treatment for methicillin-resistant *Staphylococcus aureus* (MRSA) bacteremia secondary to cellulitis of the left foot. His medical history included poorly controlled insulin-dependent diabetes mellitus and hemodialysis for chronic renal failure secondary to diabetes mellitus. Computed tomography was performed to confirm the SCJ abscess formation and bony erosions in the clavicle and adjacent sternal manubrium (Fig. 1A). Abscess was percutaneously aspired, and its cultures revealed the growth of MRSA. The patient was diagnosed with SASCJ, and treatment with intravenous vancomycin (0.65 g once every 2 days) was initiated on the day of dialysis. Surgical drainage was not performed because of the reduction in the size of the swelling. The patient did not consent for surgical management. He was discharged on intravenous vancomycin (once every 48 h for 6 weeks). The patient experienced no further swelling, erythema, or pain in the SCJ (Fig. 1B).

**Fig. 2:**
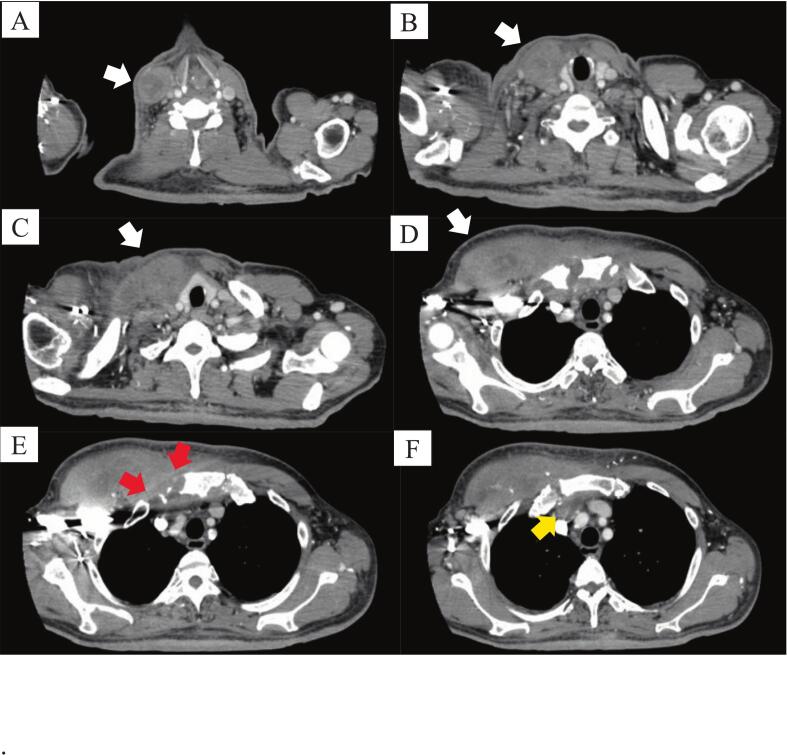
(A-F). Chest computed tomography findings. White arrows showing the cervical and anterior chest wall abscesses. Red arrows showing the destruction of the right sternoclavicular joint. A yellow arrow showing the retrosternal abscess. (For interpretation of the references to colour in this figure legend, the reader is referred to the web version of this article.)

Seven months after the last dose of vancomycin, the patient consulted his previous hospital because of severe pain in the right chest wall that had persisted for 20 days. On physical examination, the patient was found to be febrile, with a body temperature of 39.1 °C. The right SCJ exhibited swelling, tenderness, and warmth. Continuous redness was observed from the right SCJ to the right side of the neck and anterior chest wall with tenderness in the same area. Laboratory tests revealed an elevated white blood cell count (9.80 × 10^3^ μL) and C-reactive protein level (9.48 mg/dL). No neurological symptoms were noted in the right hand. Chest computed tomography revealed abscess formation in the right SCJ, right side of the neck, and anterior chest wall (Fig. 2A-F). He was diagnosed with SASCJ relapse, combined with osteomyelitis, mediastinitis, infectious myositis, and cervical and chest wall abscesses and was referred to our hospital for surgical intervention.

**Fig. 3:**
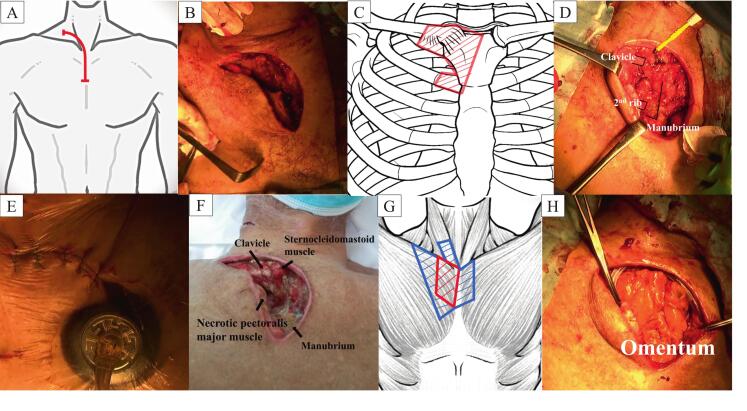
Intraoperative findings. (A-B) Skin incision. (C-D) Red diagonal lines showing the extent of resection. (E) The placement of a temporary wound vacuum device at the first operation. (F) The image of wound before 2nd operation. (G) Red and blue diagonal lines showing the extent of resection at the 1st or 2nd operation, respectively. (H) The greater omentum flap is transported into the defect. (For interpretation of the references to colour in this figure legend, the reader is referred to the web version of this article.)

Emergency surgery was performed under general anesthesia, and it precluded magnetic resonance imaging; limits assessment of spinal extension. Through a right-angle incision starting horizontally over the proximal 1/3 of the clavicle and then curving vertically along the left border of the sternum (Fig. 3A-B), the joint capsule was widely opened, and necrotic tissue was removed. Purulent discharge from the subcutaneous tissue and cervical and anterior chest wall abscesses were aspirated. After debridement of the necrotic pectoralis major muscles, the proximal 1/3 of the clavicle and half of the manubrium were excised (Fig. 3C-D). The sternal portions of the infected first and second ribs were then excised (Fig. 3C). Retrosternal abscesses were aspirated. The patient's wounds were too contaminated to consider primary closure; therefore, delayed closure was planned by placing a temporary wound vacuum device (Fig. 3E). After surgery, negative-pressure wound therapy (NPWT) was administered for infection control and wound healing.

Three weeks after the first surgery (Fig. 3F), serial re-debridement was performed in the operating room. The necrotic bilateral pectoralis major and right sternocleidomastoid muscles were also resected (Fig. 3G). Because the defect was too large for the lateral major pectoralis muscle to obliterate the residual space, we transported the greater omental flap into the deep sternal defect (Fig. 3H). A 12-cm laparotomy incision was made in the upper midline to enter the abdomen. The right gastroepiploic arteries were divided, and the greater omentum with pedicle was free and extended. The left gastroepiploic arteries was preserved during the operation to maintain blood supply to the free greater omentum. We dissected the greater omentum to the required length of 25 cm and moved it upward into the anterior chest wall defect. We ensured that there was no noticeable tension once the greater omentum filled the cavity.

Postoperatively, the patient remained afebrile. The patient was discharged on postoperative day 6, without restrictions on shoulder movement at discharge. Antibacterial medication was continued for 6 weeks based on culture and sensitivity results. The patient has been relapse-free for 1 year and has full range of motion in his shoulder and excellent arm function.

## 3 Discussion

Septic arthritis refers to bacterial invasion of a joint. It occurs in approximately 2–6 cases per 100,000 people annually [8]. Untreated septic joints lead to a risk of 10 % mortality and >30 % morbidity [9,10]. The consequences of septic arthritis include abscesses, osteomyelitis, and myositis as the infection spreads from the joint to other locations. In the case of SASCJ, timely identification and immediate intervention are crucial because SASCJ may lead to serious complications, such as osteomyelitis [11], chest wall abscess [2], mediastinitis [3], or myositis [12]. The pathogenesis of SCJ infections is not well understood. Because *S. aureus* is the most common causative organism, hematogenous spread is a possible route of infection [1,5,13]. In the present case, SASCJ developed during treatment of MRSA sepsis secondary to left-foot cellulitis.

Clinical management is controversial and is based on several relatively small case series [14]. Strategies range from surgical intervention [3,5] to conservative treatment with antibiotics alone [6]. Several confirmed cases of SASCJ, including osteomyelitis, have been successfully treated with antibiotic therapy alone [15]. However, the present case of osteomyelitis relapsed 7 months after conservative medical management and led to life-threatening complications, such as cervical and chest wall abscesses, mediastinitis, and retrosternal abscess. Surgical management should be considered in patients with osteomyelitis and bone destruction [16].

**Fig. 4:**
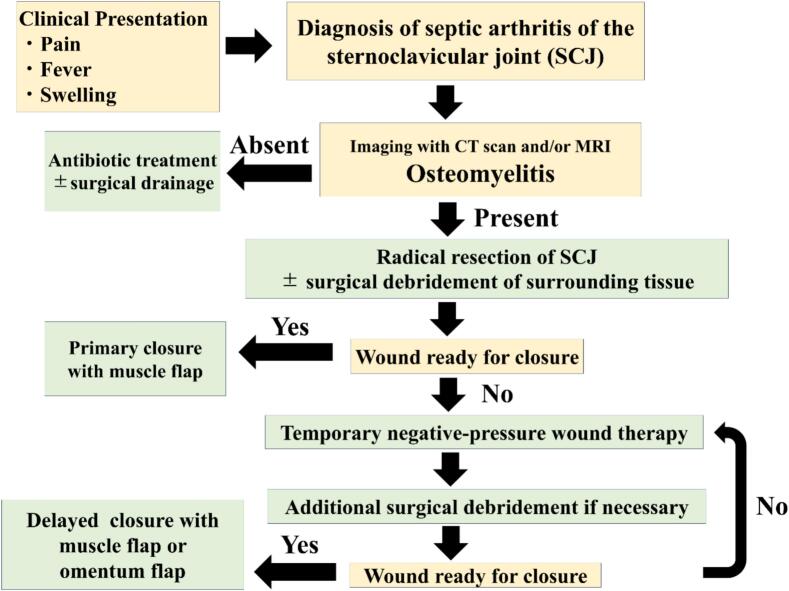
Our proposed treatment algorithm.

Surgical strategies also range from simple incision and drainage [17,18] to radical resection of the SCJ, with or without muscle transposition [3,5]. Ota et al. reported that limited surgical intervention (incision and drainage) is effective [17]. However, Song et al. from the University of Pennsylvania observed an 83 % failure rate in patients treated using this approach [19]. Glinski et al. [20] reported that en bloc resection of the SCJ is preferred for the definitive treatment of SASCJ, especially in cases of osteomyelitis. Our institution has adopted an aggressive surgical approach to manage this disease, with radical SCJ resection in cases of osteomyelitis. Our treatment strategy for advanced SASCJ cases is illustrated in Fig. 4.

Debridement of the necrotic tissue surrounding the SCJ is required in cases of soft tissue infections near the joint. If initial debridement leads to clean surfaces, one-stage closure is possible. Otherwise, a secondary closure after NPWT is required. In particular, NPWT has been shown to promote rapid granulation of large defects, enabling definitive wound closure. According to Schreiner et al., the combination of NPWT and instillation improves bacterial eradication and shortens the duration of wound care [21]. Readiness for closure of a delayed wound can be assessed subjectively by the development of granulation tissue and clearance of infected and necrotic tissues. Serial re-debridement in the operating room allows delayed closure at a later date.

Ali et al. reported low recurrence rates in patients requiring SCJ resection who underwent musculocutaneous flap reconstruction [5]. The pectoralis major flap is typically the first choice because of its suitability, as it eliminates the need for patient repositioning during surgery [19]. The simple method of raising the muscle flap and low donor-side morbidity are the reasons for the preference of the muscle flap. However, defect coverage using muscle flaps may be insufficient, particularly in cases of persistent or recurrent infections. In the present case, we extensively resected both the pectoralis major muscles. Although vascularized tissue transfer was required, locoregional flaps were unavailable or not large enough to reconstruct the defect. Therefore, we performed reconstruction using an omental flap instead of a muscle flap. This is the first case report of defect coverage using an omental flap after radical debridement for SASCJ. The omental flap has become indispensable in modern plastic surgery because it can fill deep cavities. Because of its immunological capacity and anti-infection activity, it is preferred for coverage and treatment [22]. The greater omentum is a highly active tissue for wound healing. The good vascularization pattern and immunological properties of the greater omental flap build the basis for its capacity for cellular proliferation and repair function [23,24]. By transporting the greater omental flap into the deep defect, infection can be controlled and simultaneously covered [22]. This flap is an advantageous alternative when there is a need to provide coverage for large defects.

Resection of the SCJ can be associated with instability of the shoulder girdle and unstable movement [25]. Joethy et al. observed shoulder abduction limitations in three patients who had undergone long segment clavicular resection [25]. However, functional impairment following SCJ resection is reportedly minimal in most cases [1,19,26]. For example, Ross et al. found no movement restrictions in cases involving resection of the medial one-third of the clavicle, one-half of the manubrium, and the first and second costal cartilages [1]. Song et al. [19] suggested limiting manubrium resection to one-half to preserve SCJ stability. Based on these studies, limiting manubrium resection to one-half and clavicular resection to the medial one-third is generally recommended [1,25]. During the current patient's last visit 1 year after surgery, he had full range of motion in his shoulders and excellent arm function.

## 4 Conclusion

Radical resection of the entire SCJ should be the preferred management strategy for patients with SCJ infections, especially in cases of osteomyelitis. Although the pectoralis major flap is typically the first choice to cover the defect, we used the omental flap because the locoregional flaps were not large enough to reconstruct the defect.

## Abbreviations

SCJ: sternoclavicular joint
SASCJ: septic arthritis of the sternoclavicular joint
MRSA: methicillin-resistant *Staphylococcus aureus*
CT: computed tomography
NPWT: negative pressure wound therapy

## Author contribution

Dr. Ryo Maeda is the writer of this article and corresponding author.

Dr. Ryusei Yamada, Dr. Mayu Inomata, and Dr. Fumiaki Kawano have reviewed.

## Consent

Written informed consent was obtained from the patient for publication of this case report and accompanying images. A copy of the written consent is available for review by the Editor-in-Chief of this journal on request.

## Ethics approval

As it is a case report, ethical approval is exempted by University of Miyazaki Hospital.

## Guarantor

Dr. Ryo Maeda accepts all responsibility of this article.

## Research registration number

Not applicable.

## Funding

The authors have no competing interests to declare.

## Conflict of interest statement

All authors have read and approved the final manuscript.
